# Complex Interplay of Hormonal Signals during Grape Berry Ripening

**DOI:** 10.3390/molecules20059326

**Published:** 2015-05-21

**Authors:** Ana Margarida Fortes, Rita Teresa Teixeira, Patricia Agudelo-Romero

**Affiliations:** 1 BioISI, Faculdade de Ciências de Lisboa, Universidade de Lisboa, Campo Grande, 1749-016 Lisboa, Portugal; 2 Instituto de Tecnologia de Química Biológica (ITQB), Biotecnologia de Células Vegetais, Av. da República, 2781-157 Oeiras, Portugal

**Keywords:** grape ripening, hormonal regulation, metabolome, polyamines, transcriptome, viticulture

## Abstract

Grape and wine production and quality is extremely dependent on the fruit ripening process. Sensory and nutritional characteristics are important aspects for consumers and their development during fruit ripening involves complex hormonal control. In this review, we explored data already published on grape ripening and compared it with the hormonal regulation of ripening of other climacteric and non-climacteric fruits. The roles of abscisic acid, ethylene, and brassinosteroids as promoters of ripening are discussed, as well as the role of auxins, cytokinins, gibberellins, jasmonates, and polyamines as inhibitors of ripening. In particular, the recently described role of polyamine catabolism in grape ripening is discussed, together with its putative interaction with other hormones. Furthermore, other recent examples of cross-talk among the different hormones are presented, revealing a complex interplay of signals during grape development and ripening.

## 1. Introduction

Grape (*Vitis* species) is among the most important fruit crops with a high economic impact on the global economy. Additionally, red wines are a source of many phenolic compounds that can act as antioxidants, such as resveratrol, which exhibits cardioprotective effects and anticancer properties [1].

**Figure 1:**
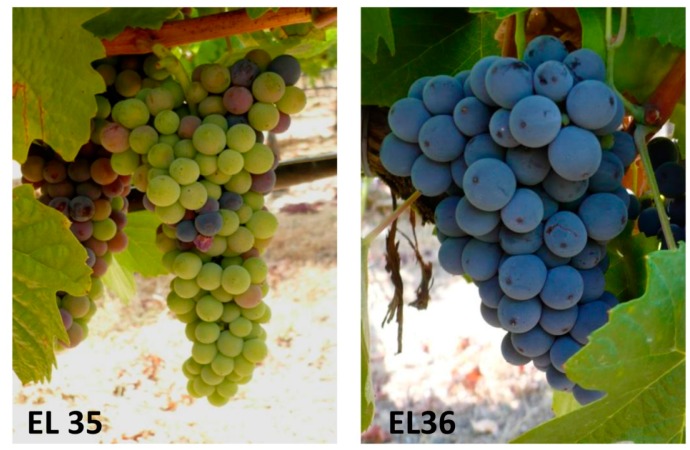
Berries of Touriga Nacional cultivar at two stages of fruit ripening: *véraison* (EL35) and berry with intermediate Brix (EL36), according to the modified E–L system [3] that is considered as the reference in this review.

**Figure 2:**
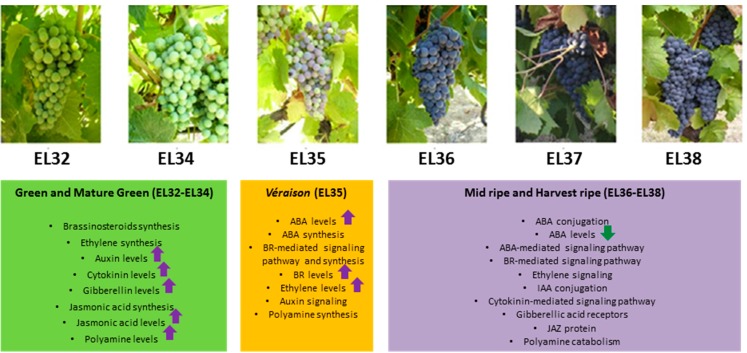
Overview of the main events involved in the hormonal control of grape development and ripening corresponding to stages EL32–EL38, according to the modified E–L system [3].

Berry development is a complex process displaying two successive sigmoid growth curves separated by a lag phase; from anthesis to ripening, grape development can be divided into three major phases [2] with more detailed descriptive designations, known as the modified E–L system, being used to define more precise growth stages over the entire grapevine lifecycle [3]. During the first phase, and considering the modified E–L system, organic acids accumulate in the vacuoles, and together with tannins and hydroxycinnamates, several precursors of phenolic compounds are synthesized (EL31–EL34). At the end of the lag phase, the short period known as *véraison* (EL35) is characterized by the initiation of sugar accumulation and the rapid pigmentation of berries by anthocyanins in red grape varieties (Figure 1 and Figure 2). High concentrations of glucose and fructose accumulate after *véraison* along with a decrease in organic acid levels and the softening of the berry [4]. The acid to sugar ratio at harvest is important for the taste of table grapes and for the sensory characteristics derived from wine grapes [4]. The third phase of berry development is known as ripening (EL36–38), when many precursors of aroma and many aroma compounds (terpenes, norisoprenoids, esters, and thiols) are synthesized [5]. Ripening is an important phase where green fruit is converted into a highly palatable, nutritionally rich, and colored fruit (Figure 1 and Figure 2) [6].

Fleshy fruits can be classified into two groups, climacteric and non-climacteric. Climacteric fruits such as tomato, banana, apple, pears, mangoes, papaya, and avocado show a concomitant increase in respiration and ethylene biosynthesis upon initiation of ripening. On the other hand, in non-climacteric fruits such as grape, citrus, and strawberry the respiratory burst and rise in ethylene production are absent at the onset of ripening. No single master switch controlling ripening initiation, such as the role played by ethylene in climacteric fruits, has yet been uncovered for non-climacteric fruits. In fact, during grape ripening, the increase in ethylene production is slight and the typical respiration peak does not occur [7]. Abscisic acid (ABA), brassinosteroids (BRs), and ethylene have been suggested to promote ripening through complex interactions, while auxin delays some ripening associated processes and also interacts with other hormones such as ABA and ethylene [8]. Moreover, a decline of polyamine contents was reported during grape ripening together with an increased catabolism of these growth regulators [9].

Albeit the amount of information already reported, the hormonal control in grape ripening, as non-climacteric fruit, is still poorly understood [10]. It is known, however, that grape berry ripening involves the integration of multiple hormonal signals; with some hormones acting as promoters and others as repressors. In particular, in non-climacteric fruits where no burst in ethylene production during ripening is observed, ABA seems to play a stronger role during ripening [11].

Several reports at transcriptional, proteomic, and metabolic levels have characterized grape ripening of specific varieties, namely Cabernet Sauvignon [12], Pinot Noir [13], Barbera [14], Corvina [15], Muscat Hamberg [16], Trincadeira [17], Touriga Nacional, Aragonês [18], and Shiraz [19]. These studies have revealed the complexity involved in the reprogramming of the transcriptome, proteome, and metabolome during grape ripening, which requires hormonal control.

In this review, we report on the current knowledge concerning hormones’ metabolism and signaling and hormonal cross-talk occurring during grape ripening.

## 2. Putative Promoters of Ripening

### 2.1. Abscisic Acid

The accumulation of ABA during grape ripening has been widely reported. In fact, it was noticed that an increase in free ABA levels around *véraison* accompanied sugar accumulation, coloration, and softening, suggesting that ABA may play a major role in controlling several ripening-associated processes of grape berry [8,10]. Transcriptomic data also support this view since it has been indicated that genes involved in ABA biosynthesis are more expressed around *véraison* [12,17]. It has also been observed that treatments with ABA during the *véraison* stage lead to an advance in red color development in Crimson Seedless grapes [20], suggesting that anthocyanin synthesis in grapes can be stimulated by exogenous ABA application. However, ABA does not necessarily sustain color acquisition since anthocyanins keep increasing throughout the ripening period, while ABA levels start to decrease at the same time [21]. In an ABA-deficient mutant in orange fruit, the coloration of the fruits skin was delayed compared with that of the wild type during maturation [22]. This phenotype was overcome by the application of ABA. Analysis of the proteome of Cabernet Sauvignon berries showed that ABA treatment leads to an up-regulation of the expression of several ripening and stress- associated proteins [23].

In addition, ABA may be able to hasten the initiation of sugar accumulation when applied before *véraison*, by stimulating the uptake and storage of sugars by berries [10]. Pan and co-workers [24] demonstrated that ABA increased the activity of both soluble and cell wall acid invertases in Kyoho berry discs, providing evidence of a link between ABA, invertase activity, and sugar metabolism. Besides grape, ABA accumulation was suggested to play a key role in the regulation of ripeness and senescence of tomato [25], and in the regulation of development and ripening of the cucumber, a non-climacteric fruit [26]. These results are indicative of the involvement of ABA in the maturation of both climacteric and non-climacteric fruits.

Furthermore, it was observed that ABA treatment reduced the tannin content of green grapes without modifying their composition, but had a positive impact on tannin biosynthesis during *véraison*, as previously demonstrated for anthocyanins, suggesting that leucoanthocyanidin reductase and anthocyanidin reductase which are enzymes involved in tannin biosynthesis were co-regulated by ABA [27].

The endogenous ABA content is determined by the dynamic balance between biosynthesis and catabolism [28] which varies among different clusters and among berries of the same cluster. In fact, ABA levels change at *véraison* among berries of the same cluster [29]. ABA biosynthesis involves crucial steps catalyzed by two plastidial enzymes, namely, 9-*cis*-epoxy-carotenoid dioxygenase (NCED) and zeaxanthin epoxidase (ZEP), whereas ABA catabolism is regulated mainly by ABA 8′-hydroxylase CYP707A1. The control of ABA levels also involves the activity of cytosolic UDP-glucosyltransferases that conjugate ABA to form the ABA-glucose ester, and the activity of β-glucosidases that release ABA [28].

Genes coding for 9-*cis*-epoxycarotenoid dioxygenases have been shown to present a peak in expression at EL35 in several cultivars [12,17]. A gene coding for zeaxanthin epoxidase (*ZEP*) has also been detected as up-regulated at EL35 stage compared to EL36 in Trincadeira, Touriga Nacional and Aragonês cultivars [17,18] in agreement with an increase in ABA levels around *véraison*. Furthermore, a gene coding for ABA 8′-hydroxylase CYP707A1 was down-regulated in the three Portuguese varieties at EL36 [18]. This suggests that during ripening ABA levels may be controlled mainly by conjugation to glucose [30] (Figure 2).

Experiments carried out in strawberry showed that a remarkable decrease in ABA content due to the down-regulation of the transcripts of a *9-cis-epoxycarotenoid dioxygenase* gene (*FaNCED1*) by virus-induced gene silencing, can significantly retard the ripening process, providing direct molecular evidence on the requirement of ABA for ripening of the strawberry, a non-climacteric fruit [31].

On the other hand, a set of genes involved in the ABA-mediated signaling pathway increased their expression at EL36 stage in three Portuguese varieties: two ABA receptors, namely, Abscisic acid receptor PYL8 RCAR3 and Abscisic acid receptor PYL9 RCAR1; Protein phosphatase 2C FSPP2C2; and VP1/ABI3 family regulatory protein [17,18]. It was previously shown that PYL8/RCAR3 has a positive role in regulating ABA signaling [32]. ABA receptors belonging to the PYR/PYL/RCAR family constitute ABA sensors able to inhibit the activity of specific protein phosphatases type-2C (PP2Cs) in an ABA-dependent manner [33]. These results suggest that later in ripening, ABA synthesis is not induced, but instead, ABA-regulated processes (e.g., abiotic stress responses) are activated. Recently, it was shown using an RNAseq approach that berry maturation was accompanied by enhanced stress related metabolism, such as ABA in Shiraz. This cultivar is more susceptible towards environmental cues than Cabernet Sauvignon [34]. This up-regulation of ABA related genes in Shiraz was associated with enhancement of varietal specific anthocyanin accumulation [34].

### 2.2. Brassinosteroids

Brassinosteroids (BRs) are steroidal plant hormones essential for normal plant development. They have also been described as ripening promoters in non-climacteric fruits such as grapes. Genes involved in brassinosteroid biosynthesis and sensing have been cloned and their transcriptional profile characterized during grape development in Cabernet Sauvignon [35]. They revealed transcript accumulation patterns that were consistent with a dramatic increase in endogenous BR levels (castasterone and its direct precursor 6-deoxocastasterone) observed at the onset of fruit ripening (Figure 2). Moreover, applications of Epi-BL hastened berry ripening whereas an inhibitor of BRs biosynthesis, brassinazole, delayed ripening [35]. The effects of the epi-BL and brassinazole treatments were evident in both the appearance of skin coloration and the final sugar levels in ripe berries. Recently, the role of brassinosteroids in the regulation of anthocyanin biosynthesis during ripening of grape berries was analyzed in more detail [36]. The authors concluded that the effect of BR on downstream genes of anthocyanin biosynthesis was more effective than that on upstream genes, leading to enhancedfruit coloration.

In the Pinot Noir cultivar, it was reported that the transcript abundance of a gene coding for *VvBR6OX1*, which converts 6-deoxocastasterone to castasterone, the only bioactive brassinosteroid detected in grape, peaked just before *véraison*, declining thereafter [13]. In the Trincadeira cultivar this gene was down-regulated at EL35 and EL36 compared to EL32 [17]. A negative correlation in other species was noticed between *VvBR6OX1* transcript abundance and the amount of the corresponding enzyme substrate [10]. On the other hand, BRs may also be involved in early fruit development, as occurs in strawberry [37].

In previous studies, a set of several genes was down-regulated in three Portuguese cultivars at ripe stage when compared to *véraison*, and codify for BRI1 protein, BRI1-KD interacting protein, and BKI1 (BRI1 kinase inhibitor 1). By contrast, *BRL3* gene (*BRI1-like 3*) was detected to be up-regulated at ripe stage. The same pattern of expression was seen for the BSU1-like protein 3 BSL3, a protein involved in the brassinosteroid-mediated signaling pathway [17,18]. Brassinosteroids are perceived by the plasma membrane-localized leucine-rich-repeat-receptor kinase BRI1. *BRL1* and *BRL3* encode functional BR receptors that bind brassinolide in Arabidopsis [38]. The putative receptor gene, *VvBRI1*, was expressed throughout berry development with a peak in transcript levels shortly after *véraison* [35].

The expression of BR receptors during ripening puts in evidence the role of BRs in this stage of berry development. Interestingly, the transgenic up-regulation of the signal transduction pathway of BRs in tomato leads to increased content in carotenoids and soluble solids in ripe fruit [39]. However, there is a need for more studies addressing the complex process of BR biosynthesis and signaling during grape development, in particular the inclusion of more time points around *véraison* and around different seasons. Previously, differences in perception of brassinosteroids were suggested to be due to different climatic conditions or eventually due to tissue specific expressions [17].

### 2.3. Ethylene

During grape ripening, the increase in ethylene production around *véraison* is slight and the typical respiration peak does not occur [7]. We have previously reported the down-regulation at EL36 *vs.* EL35 of genes coding for ACC synthase and ACC oxidase, which are enzymes involved in ethylene biosynthesis [9]. In addition, the peak of expression was observed at EL32. In other grapevine cultivars the peak in ACC oxidase transcript accumulation occurred immediately before *véraison* or in green berries [12,13]. Nevertheless, one gene coding for ACC oxidase was found to increase its expression at EL36 and EL38 by microarray analysis, which was further confirmed by real-time qPCR [9]. The results suggest that the peak occurs before *véraison* but some isoforms of ACC oxidase may be active following *véraison*. In watermelon, a non-climacteric fruit, a homolog of ACC oxidase was also induced in the ripening stages [40].

The application of 2-chloroethylphosphonic acid (2-CEPA), an ethylene-releasing compound, on berries at *véraison* led to an increase in the concentration of several anthocyanin derivatives in Cabernet Sauvignon berries without affecting berry weight [41]. On the other hand, treatments with 1-methylcyclopropene (1-MCP), a specific inhibitor of ethylene receptors, decreased berry diameter and anthocyanin content and increased acidity in Cabernet Sauvignon berries [7]. However, the effect of such treatments strongly depends on the developmental stage of the berries at the time of application suggesting that there is modulation of ethylene perception and/or signaling [10].

Regarding this, the transcript levels of some grape ethylene receptors were reported to change during berry development [12,17,42]. This is in agreement with decreased ethylene levels during ripening since ethylene binding has been proposed to inhibit receptor function [43].

In our previous survey across three Portuguese varieties, we found as putative positive markers of ripening genes coding for ethylene-responsive transcription factor 9, AP2-like ethylene-responsive transcription factor and ethylene-responsive element binding factors [17,18]. ERF belongs to a large family of APETALA2-domain containing transcription factors that bind to promoters of many ethylene inducible genes. These results provide evidence that the ethylene-mediated signaling pathway is active during grape ripening, as seems to occur in pepper, another non-climacteric fruit [44]. Recently, a significant induction of ethylene signaling and flavor pathways in the skin was detected at late stages of berry ripening [45]. In this work, the transcript abundance of many ERF transcription factors was significantly affected by berry ripening and the transcript abundance of a unique clade of ERF6-type transcription factors was correlated with the transcript abundance of terpene synthases and lipoxygenases involved in flavor formation.

Additionally, it was detected an up-regulation at EL36 in the three Portuguese varieties of a gene coding for a MAP3K protein kinase [17,18]. The Arabidopsis MPK3 is involved in the regulation of the ethylene response pathway [46].

## 3. Putative Inhibitors of Ripening

### 3.1. Auxins

Auxins are involved in many aspects of plant development. In grape berries, developmental changes in auxin levels are still a matter of debate. Some studies suggest that indole-3-acetic acid (IAA) concentrations are high during the early fruit developmental stages, declining afterwards and remaining low throughout the rest of berry development [47] (Figure 2). However, IAA concentrations have also been claimed to be low and relatively constant throughout berry development, including during *véraison* [10,35]. Nevertheless, low auxin levels seem to be required at the onset of grape ripening, which is associated with an increase in the conjugated form of indole acetic acid [48]. In fact, auxin delays increase in berry size, sugar accumulation, and anthocyanin content [49,50,51]. Treatment with NAA at pre-*véraison* suppressed accumulation of anthocyanins, and the application of BTOA, an auxin-like compound, to Shiraz berries prior to *véraison* delayed ripening and altered gene expression [49,51]. Recently, it was suggested that that NAA application affects the natural decline of IAA in advanced berries and delays the ripening, thereby reducing variance between under-ripe and riper berries of the same cluster [29].

It is generally accepted that auxin plays a role in fruit growth but delays the ripening-related processes in climacteric and non-climacteric fruits [6,8].

An important process for the regulation of auxin levels in grapes is the inactivation of IAA by its conjugation [48]. Two genes coding for indole-3-acetate *beta*-glucosyltransferase putatively involved in auxin inactivation by conjugation were down-regulated at EL36 in three Portuguese varieties [17,18]. Another gene coding for Auxin-amidohydrolase precursor putatively involved in IAA homeostasis and auxin activation by conjugation hydrolysis was also down-regulated. Furthermore, a gene coding for IAA-amino acid hydrolase 1 (ILR1), also related to auxin activation by conjugation hydrolysis was up-regulated at EL36 [17,18]. These results clearly demonstrate that the fine regulation of the pool of IAA conjugates during grape ripening is common to several cultivars.

Moreover, a core set of several genes involved in auxin signaling has been found to be differentially expressed in three cultivars, namely, the down-regulation at EL36 of genes coding for two Auxin response factors (ARF), 4 and 9 [17,18]. ARFs are transcription factors that regulate the expression of auxin response [52]. Recently, *ARF4* was suggested to mediate the response to auxin changes during the grape berry ripening initiation, and likely to be involved as a negative regulator of the ripening-related changes in the pericarp during *véraison* [53]. In addition, two genes coding for IAA9 and IAA16 were down-regulated at EL36 [17,18]. These genes have been identified as rapidly induced auxin response genes [52]. Regarding auxin responsive genes, one can be considered a positive marker of ripening of three Portuguese varieties (Auxin-responsive SAUR29), while another can be considered a negative marker (Auxin-responsive SAUR12)*.* Additionally, genes involved in auxin transport were up-regulated (AUX1 auxin influx carrier protein, and Auxin transporter protein 2) or down-regulated (Auxin efflux carriers, and *PIN1*) [17,18]. Little is known about auxin transport and signaling in ripe fruits but the topic certainly deserves attention.

### 3.2. Cytokinins

Cytokinins are involved in berry set as well as in growth; higher levels are often found in the flesh of immature fruits, but decrease rapidly at around the time of *véraison* and are kept low in maturing and mature fruits [47,54] (Figure 2). The profile is consistent with a role in early fruit development in particular cell division as well as in inhibition of ripening. Moreover, the use of cytokinin-like compounds such as forchlorfenuron, are commonly applied in many vineyards worldwide, mainly for production of table grapes in order to achieve optimal berry size and improved yield. This treatment leads to increased content in condensed tannins and has a negative impact on anthocyanin content [55,56,57].

In a previously mentioned work, the down-regulation of a gene coding for Cytokinin dehydrogenase 5 precursor at EL36 in comparison with EL35 in three Portuguese varieties [18] was observed. This gene is putatively involved in cytokinin degradation. Deluc *et al.* [12] and Fortes *et al.* [17] have also reported a decrease in the transcript abundance of this gene over berry development. Furthermore, a gene coding for purine permease 1 (PUP1) putatively involved in cytokinin transport was also down-regulated at EL36 [18]. Regarding the cytokinin-mediated signaling pathway, two genes: ARR3 type-A and ARR1 type-B, were identified as positive markers of ripening in three Portuguese varieties [17,18]. Type-B response regulators (ARRs) are DNA-binding transcriptional activators required for cytokinin responses whereas the type-A ARRs act as repressors of cytokinin-activated transcription [58].

### 3.3. Gibberellins

Gibberellins (GAs) are regulators of a vast number of processes during plant development, such as cell division and cell expansion. Application of gibberellin is widely used in the early stages of seedless berry development to increase berry size and, therefore, its economic value. In grapevine, biological active concentrations of GAs were found to be high in the flowers and during early berry development, but dropping to lower levels throughout the subsequent berry development [35,47] (Figure 2). Giacomelli and co-workers [59] noticed the accumulation of bioactive GA_1_ in opening grapevine flowers, whereas at later developmental stages only GA_4_ was detected in the setting fruit. Moreover, the pool of bioactive GAs is controlled by a fine regulation of the abundance and localization of GA oxidase transcripts. Previously, Fortes *et al.* [17] showed that two genes coding for gibberellin oxidase were up-regulated at EL35 and EL36 when comparing to EL32, but others were down-regulated at the same stages, making it difficult to understand how the metabolism of gibberellins is processed during ripening. In a previous study, a gene coding for Gibberellin 20 oxidase 2 was down-regulated at EL36 in three grape varieties [18] and can be therefore considered as a putative negative marker of berry ripening.

The transcript abundance of two putative Gibberellic acid receptors, GIDL1 and GIDL2, was shown to increase during development of Cabernet Sauvignon grapes [12]. In Trincadeira grapes collected in the 2007 and 2008 seasons [17], an up-regulation of a gene coding for GID1L1 at ripe stage comparing to green stage (EL36 *vs.* EL32) was observed and a higher transcript abundance in 2007 when the ripe berries were larger was noticed. The same gene was up-regulated at EL36 *vs.* EL35 in three varieties [18]. Interestingly, the transcript abundance was lower in Touriga Nacional which produces smaller berries [18]; thus, this may be related to the involvement of gibberellins in cell enlargement. Despite these results there is still little evidence to suggest that GAs are directly involved in the control of berry ripening [10].

### 3.4. Jasmonic Acid

There is lack of strong evidence that links endogenous jasmonates to the control of fruit ripening. However, in non-climacteric fruits such as strawberry and grape, it has been reported that the jasmonates’ levels are high in early development, decreasing to lower values in ripe fruits, and this may enable the onset of ripening to occur [60,61]. In addition, mRNAs involved in the biosynthesis of jasmonic acid were reported to be less abundant at EL35 and EL36 compared to EL32 [17].

In grapevine, treatment of cell cultures with methyl jasmonate increased the production of the stilbene *trans*-resveratrol [62]. Experiments *in vitro* of methyl jasmonate applications on green strawberry fruits showed acceleration of ripening with an increase in softening and anthocyanin content along with an increase in the expression of several phenylpropanoid-related genes, in particular those associated with anthocyanin biosynthesis [63].

The gene coding for jasmonate *O*-methyltransferase or *S*-adenosyl-l-methionine:jasmonic acid carboxyl methyltransferase was down-regulated in ripe fruits of three grape varieties [18]. This gene is putatively involved in volatile methyl jasmonate synthesis. In strawberry, it was noticed that jasmonic acid carboxyl methyltransferase transcript levels decreased steadily during fruit ripening in accordance with methyl jasmonate levels, as well as the expression levels of jasmonic acid biosynthetic genes [64].

Also, in our previously published data, the gene coding for the jasmonate ZIM domain-containing protein 8 was identified as a putative positive marker of ripening (Figure 2) since it presented the same transcriptional profile in three grape varieties [18]. In Arabidopsis, jasmonate ZIM-Domain (JAZ) proteins have been reported as repressors of jasmonic acid signaling [65]. Taking into consideration all these results, it seems that jasmonates play a role in the early stage of fruit development and in the onset of ripening, possibly through interaction with other growth regulators, as will be discussed in another section.

### 3.5. Polyamines

Polyamines are growth regulators that have been implicated in several developmental processes as well as in biotic responses. Polyamines have also been reported to be inducers of flowering, promoters of fruitlet abscission, and involved in fruit set in grapevine [66]. However, the information related to fruit ripening is very scarce.

In grapes, the content in free polyamines decreases during ripening, reaching the lower value at the last stages of maturity [9,67,68]. The same holds true for climacteric fruits such as apple [69], avocado [70], and peach [71]. Due to these reasons, polyamines have been reported to be important during early fruit development, eventually associated with cell proliferation [67,72]. However, studies on gene expression during grape ripening have revealed that genes coding for arginine decarboxylase, spermidine synthase, and spermine synthase, involved in the synthesis of polyamines, increased their transcript abundance around the onset of ripening and remained high in ripe fruits [12,17]. Moreover, complementary metabolic profiling performed on Trincadeira, Touriga Nacional, and Aragonês cultivars showed an accumulation of GABA and l-arginine during grape ripening [73], suggesting that the metabolism of polyamines is active during ripening. In particular, GABA is a product of catabolism of polyamines due to diamine oxidase activity [9].

This fact prompted us to deepen the study of polyamine metabolism during grape ripening [9]. In this work, we performed a detailed analysis of the polyamine pathway using transcriptomic approaches complemented by HPLC analysis of polyamine contents, as well as enzymatic activity assays of the polyamine catabolic enzymes (Diamine Oxidase–*CuAO* and Polyamine Oxidase–*PAO*).

The results showed an up-regulation of a gene coding for arginine decarboxylase during grape ripening in three grapevine varieties in two consecutive years [9]. However, free and conjugated polyamines presented a strong decrease comparing to early stages of development, in particular putrescine and spermidine. The changes in polyamines content during grape ripening was accompanied by up-regulation of genes coding for diamine oxidase and polyamine oxidase, together with a significant increase in their enzymatic activity and in the hydrogen peroxide content (a product of polyamine catabolism). These results provided, for the first time, strong evidence of a role of polyamine catabolism in grape ripening [9]. Polyamine catabolism may constitute a source of reactive oxygen species as H_2_O_2_ to signal downstream stress defense events towards abiotic and biotic stress factors, which ripe fruits are more susceptible to due to sugar accumulation.

The increase in the expression of genes coding for *CuAO* and *PAO* varied among varieties, the peak being in their expression detected either at EL36 or EL38, making it difficult to establish them as markers of a particular stage. However, they can undoubtedly be considered as positive molecular markers of ripe fruits [9]. On the other hand, genes coding for ornithine carbamoyltransferase and glutamate *N*-acetyltransferase can be considered as putative negative markers of EL36 stage, according to previous work [18]. Glutamate *N*-acetyltransferase is involved in the synthesis of ornithine and arginine, but the major pathway of polyamine in grape is via arginine and agmatine [72].

To further clarify the role of polyamine catabolism in grape ripening experiments using guazatine, a potent inhibitor of polyamine oxidase activity, were conducted with in-field grown berries of cultivar Trincadeira [68]. This variety was the one presenting a higher content in polyamines at EL32 [9]. The results suggested that in guazatine-treated berries transport mechanisms were impaired, leading to a strong dehydrated phenotype. There was more content in putrescine, and in the osmotically active solute proline, in guazatine- treated samples. Genes coding for *CuAO* and *PAO* were down-regulated in guazatine-treated samples at EL38 whereas activity assays showed inhibition only of polyamine oxidase but not of diamine oxidase. This inhibition caused impact on carbohydrate, hormone, and secondary metabolisms. In particular, it affected terpenoid metabolism with possible implications in aroma development and fruit quality [68]. Recently, the increase in polyamines in green grapes due to infection with *Botrytis cinerea* was suggested, based on the transcriptional profiling of genes involved in polyamine biosynthesis [74]. Moreover, GABA was only detected in infected grapes, suggesting an active polyamine catabolism in non-ripe but infected grapes that may be involved in the observed impact on aroma development [74].

Transgenic tomato lines transformed with yeast SAM decarboxylase showed an accumulation of spermidine and spermine and revealed that these higher polyamines influence multiple cell pathways in climacteric fruit ripening [75]. Our results concerning grapes provide compelling evidence that polyamines, and, in particular, polyamine catabolism, play an important role in non-climacteric fruit ripening (Figure 2). More research is needed to verify this role in other fruits, especially climacteric fruits, where an ethylene burst occurs and may compete with polyamines, since they share *S*-adenosylmethionine as a common precursor.

## 4. Hormonal Crosstalk

The very large number of examples of possible crosstalk amongst hormones makes it impossible to explore all of them in this review. Therefore, only some examples are reported in this section.

Reports about changes in the profile of the grape transcriptome during ripening, either using microarrays or RNAseq technology, have been a fundamental tool to gather knowledge on the cross-talk between the different signaling pathways [12,13,15,17,19].

Functional interaction and synergism between ABA and ethylene at the onset of grape berry ripening has been found [42]. The trace ethylene is active when the expression of *VvNCED1* increases gradually in the *véraison* [76]. In order to test whether trace endogenous ethylene may induce the expression of *VvNCED1* and also the accumulation of ABA, Sun and co-authors [42] treated grape clusters one week before *véraison* with both NiCl2 and 1-MCP to double block ethylene biosynthesis and ethylene signal. The treatment suppressed the transcription of *VvNCED1* and the production of ABA. Treatment with ABA could relieve the double block and restore the fruit ripening course. However, after harvest, the same double treatment could not suppress the transcription of *VvNCED1* and the accumulation of ABA, and also could not inhibit the start of fruit senescence. So the effects of ethylene inhibition are different according to the developmental stage of the berry. The authors concluded that both ethylene and ABA are likely to be important and their interplaying may be required to start the process of berry ripening. In climacteric fruits, there is also evidence suggesting that ABA is an inducer of ripening by promoting ethylene biosynthesis in tomato [25] and in banana [77].

While ethylene seems to induce ABA, auxin negatively regulates ABA-induced ripening processes. Treatments of Shiraz berries with benzothiazole-2-oxyacetic acid (BTOA), an artificial auxin, caused a two week delay in the increase in ABA content [49]. Transcriptomic analyses of berries treated with naphthaleneacetic acid (NAA) at pre-*véraison* showed strongly a delay in ripening inception, with down-regulation of genes involved in flavonoid biosynthesis and cell expansion. This delay was accompanied by an increase in expression of ethylene biosynthetic genes and a decrease in expression of genes involved in ABA biosynthesis and perception [51]. Furthermore, interactions between ethylene and auxin were recently reported to be fundamental to the control of berry ripening [78]. Exposure of *ex planta*, pre-ripening berries to the ethylene biosynthesis inhibitor aminoethoxyvinylglycine led to a decrease in the levels of IAA and IAA-Asp, whereas auxin biosynthesis was stimulated by the ethylene-releasing compound Ethrel, causing also a delay in ripening. In non-treated grape berries, the increase in expression of the putative auxin biosynthesis and conjugation genes was preceded by high expression levels of the ethylene biosynthesis.

As previously mentioned, gibberellins application is widely used in the early stages of seedless berry development to increase berry size. Recently, it was shown using RNA sequencing, that exogenous gibberellins (GA_3_) induced multipoint cross talk with auxin, cytokinin, brassinosteroid, ABA, and ethylene [79]. The GA_3_ treatment seemed to induce an auxin-perception feedback regulation and down-regulation of the majority of genes coding for cytokinin *trans*-hydroxylase involved in *trans*-zeatin biosynthesis. GA_3_–ethylene cross talk was mainly reflected by the conditional regulation of the abundance of ERFs in the grape berries. In addition, it was observed down-regulation of genes involved in ABA biosynthesis and signaling.

While gibberellins inhibit ABA biosynthesis, polyamines seem to stimulate increased synthesis of ABA in guazatine-treated grapes [68]. Moreover, genes involved in ABA signaling were up-regulated due to this treatment, which can be considered an abiotic stress. A plethora of data on the analysis of mutant plants and transcript profiling suggest a positive feedback mechanism between putrescine and ABA; both hormones are likely to reciprocally promote each other’s biosynthesis under stress to enhance the plants’ adaptive potential [80,81]. Furthermore, genes coding for proteins involved in ethylene and jasmonate signaling appeared mainly in guazatine-treated samples. Functional interaction and synergism between ABA and ethylene during grape berry ripening and after harvest has been shown [42]. The results also suggested that interaction between these two hormones may occur in response to guazatine treatment, which apparently leads to premature withering of grapes [68].

## 5. Conclusions and Future Prospects

The hormonal control of grape ripening is extremely complex involving the interplay of several growth regulators. Furthermore, individual ripening processes themselves, such as fruit color and softening, sugar accumulation, and aroma development, may be under specific hormonal control [11]. In this review, we discussed certain hormones as promoters or inhibitors of grape ripening, but this distinction is often not clear and depends on the developmental stage associated with exogenous application. One of the limitations of grape is the difficulty in conducting reverse genetic experiments to analyze gene function or obtaining new phenotypes. Neither stable nor transient expression protocols have been efficiently established in grape, and ripening mutants are lacking. Therefore, there is no evidence for any direct interactions between hormone pathways during berry development. The development of techniques, such as fruit agroinfection, applied to grape will certainly contribute to establish grape as a main model system for fruit functional genomics, as it currently stands for tomato. The understanding of the hormonal control of grape ripening will certainly benefit from the availability of such tools.
